# Rapid Detection of Coronavirus (COVID-19) Using Microwave Immunosensor Cavity Resonator

**DOI:** 10.3390/s21217021

**Published:** 2021-10-23

**Authors:** Dalia M. Elsheakh, Mohamed I. Ahmed, Gomaa M. Elashry, Saad M. Moghannem, Hala A. Elsadek, Waleed N. Elmazny, Nelly H. Alieldin, Esmat A. Abdallah

**Affiliations:** 1 Microstrip Department, Electronics Research Institute (ERI), El Nozha 11843, Egypt; miahmed@eri.sci.eg (M.I.A.); Gomaa.M.Ashry@eri.sci.eg (G.M.E.); hhelsadek92@gmail.com (H.A.E.); esmataa2@hotmail.com (E.A.A.); 2 Electrical Department, Faculty of Engineering and Technology, Badr University in Cairo, Badr 11829, Egypt; 3 Botany and Microbiology Department, Faculty of Science, Al-Azhar University, Cairo 11651, Egypt; saadmoghannem@azhar.edu.eg; 4 Holding Company for Biological Products and Vaccines (VACSERA), Dokki, Giza 12654, Egypt; waleednelmazny@gmail.com; 5 National Cancer Institute, Cairo University, Giza 12613, Egypt; nelly26660@gmail.com

**Keywords:** micro-immunosensor, electromagnetic sensing, diagnostics, COVID-19, coronavirus, pocket vector network analyzer (VNA), microwave cavity resonator (MCR), ELISA, real-time polymerase chain reaction (RT-PCR)

## Abstract

This paper presents a rapid diagnostic device for the detection of the pandemic coronavirus (COVID-19) using a micro-immunosensor cavity resonator. Coronavirus has been declared an international public health crisis, so it is important to design quick diagnostic methods for the detection of infected cases, especially in rural areas, to limit the spread of the virus. Herein, a proof-of-concept is presented for a portable laboratory device for the detection of the SARS-CoV-2 virus using electromagnetic biosensors. This device is a microwave cavity resonator (MCR) composed of a sensor operating at industrial, scientific and medical (ISM) 2.45 GHz inserted in 3D housing. The changes of electrical properties of measured serum samples after passing the sensor surface are presented. The three change parameters of the sensor are resonating frequency value, amplitude and phase of the reflection coefficient |S_11_|. This immune-sensor offers a portable, rapid and accurate diagnostic method for the SARS-CoV-2 virus, which can enable on-site diagnosis of infection. Medical validation for the device is performed through biostatistical analysis using the ROC (Receiver Operating Characteristic) method. The predictive accuracy of the device is 63.3% and 60.6% for reflection and phase, respectively. The device has advantages of low cost, low size and weight and rapid response. It does need a trained technician to operate it since a software program operates automatically. The device can be used at ports’ quarantine units, hospitals, etc.

## 1. Introduction

Wuhan, on 30 December 2019, reported a new strain of the coronavirus family that caused acute respiratory syndrome (SARS), which may result in death. The strain spread quickly and became a major global public health concern with over 4.5 million new cases reported in the last week of April 2020 [1,2]. The total number of people to have caught this disease exceeds 114 million, while the death toll currently stands around four million [3,4,5,6]. As a consequence, this pandemic has led to the contraction of all economic activities all over the world [7]. Due to the dramatic increase of patients, there is a serious demand for rapid diagnostic methods and rapid detection of coronavirus.

At this time, there is no rapid detection with good accuracy of COVID-19 in less than 6 h [8]. However, time is a very important factor in limiting the spread of pandemics [9,10]. Biosensors are important tools that can be integrated in a variety of applications. The need for high speed, sensitivity and accuracy of analytical measurements have led to a high interest in developing many types of sensors as diagnostic tools [11,12]. These sensors use different parameters such as enzymes, cell receptors, antibodies and nucleic acid. The new sensors should be cost effective as compared to readily existing ones. New biosensors should have a small size, high sensitivity and rapid response. Manufacturing a local portable diagnostic biosensor will have a great impact on the local market and Egyptian health care [13,14,15,16]. Also, this will provide rapid detection and diagnosis of those contagious with the virus, especially in rural areas, to limit the spread of virus. Different techniques have been investigated and used for virus detection with nanomaterial [17] facile biosensor screening [10]. Table 1 [14] presents a comparison between our proposed MCR device and different diagnostic techniques for COVID-19.

Biosensors are an attractive method for label-free capability of monitoring bacteria and viruses [19]. A microwave resonator is extensively used nowadays as a contactless and non-invasive method for virus detection with a transmission coefficient |S_21_| [20] resonant frequency and quality factor [21,22].

**Figure 1:**
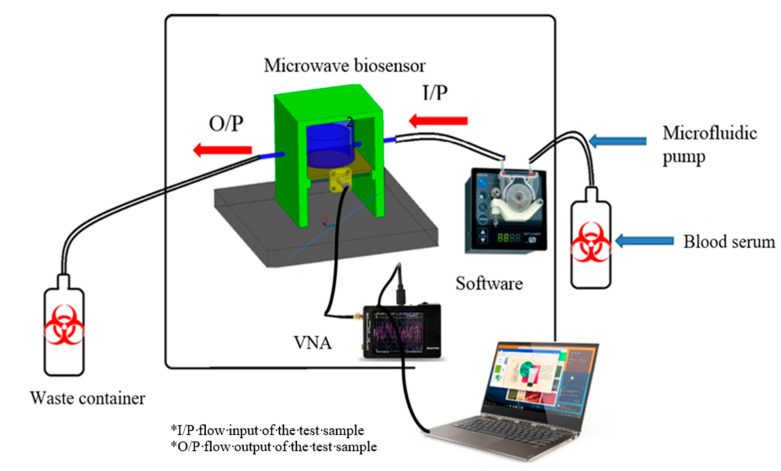
Complete layout of the proposed device.

The main idea behind this device was published early in [18]. The system consists, as shown in Figure 1, of the following main components: microfluidic pump, pocket vector network analyzer, 3D dimension housing, waste containers with silicon tubes, and control software. It should be noted that our gold reference is polymerase chain reaction (RT-PCR) as a quantitative and qualitative nucleic acid [9].

This paper is organized as follows: Section 1 gives an introduction, presenting the importance of this device as well as existing systems on the market. Section 2 introduces the sensor’s design and the layout of the system, while Section 3 details the immobilization of corona antibodies on the sensor gold surface. The system’s integration and assembly are introduced in Section 4. A sample of measured results for detecting the existence of coronaviruses is presented in Section 5, while the statistical analysis and software package is introduced in Section 6. Section 7 gives the measured samples and measurement methods, and Section 8 concludes the paper.

The proposed sensor depends on the bond that exists between the antigen and the antibody. It should be noted that most sensors depend on this biological idea used by blood banks worldwide, like ELISA, for example. Serological techniques normally depend on a color development reaction that occurs due to specific binding between antigens and antibodies followed by enzymatic activity, which are coupled to the antibodies used (this step usually is complicated and time consuming, needing a highly equipped laboratory and well-trained technicians) [23,24,25]. In this paper, the device measures the change in the electromagnetic properties of the sensor due to the specific binding between antibodies immobilized at the chip surface and antigen directly through the layer of growth over the sensor surface in real-time. This method (sensor) has the advantage of being used for many viruses and bacteria since it depends only on the type of immobilized antibodies on the sensor so it has multiple specificity. Other types of methods can be used for only one pathogen at a time.

## 2. Sensor Design and Layouts

**Figure 2:**
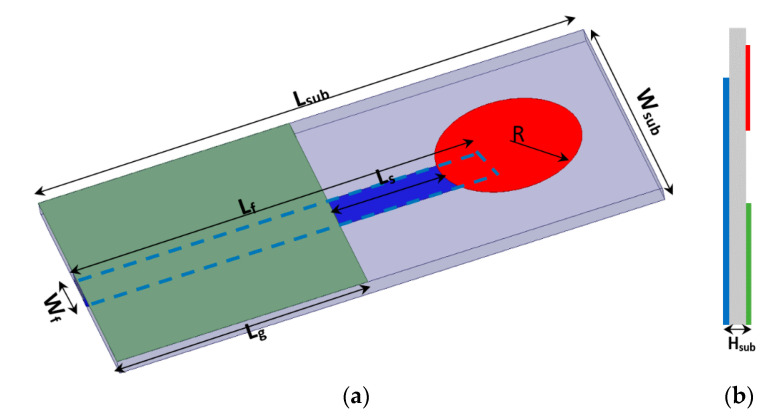
(**a**) 3D geometry of the proposed sensor antenna, and (**b**) side view.

**Figure 3:**
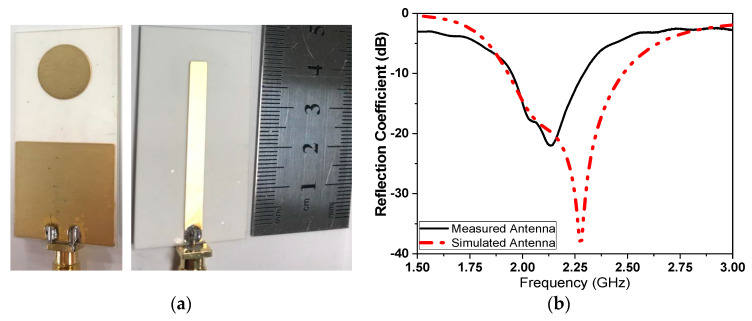
(**a**) Fabricated sensor with gold-plated surface, and (**b**) simulated and measured comparison of the reflection coefficient.

The proposed micro immune-sensor is a planar microstrip antenna. A gold layer is deposited above the antenna to prevent an interaction between the surface of the antenna and any liquids (blood serum). The performance of the antenna—namely, the reflection coefficient (magnitude and phase)—is measured using a pocket VNA. The main idea depends on the fact that any changes in the sensor’s parameters are attributed to changes in the electrical properties such as conductivity, dielectric constant, etc. [25,26,27]. There are many sensors that depend on the bio-recognition process. In these types of sensors, the antibodies (receptor) are immobilized on the sensor surface, while antigen will flow over the sensor (the detection region) to allow for the interaction between the antigen and antibodies that already exist on the sensor’s surface. In this case, a very thin layer of dielectric material with certain permittivity exists on the surface of the sensor, causing changes in various parameters such as the frequency and the reflection coefficient (magnitude and phase) [28,29,30,31]. The sensor is designed to be extremely sensitive to changes in permittivity on its surface, which are measured. A low-profile microstrip disc antenna with a microstrip line-proximity coupling feed is designed and simulated by using 3D full-wave electromagnetic simulation from a High-Frequency Structure Simulator (HFSS) ver. 14. The circular patch antenna, as shown in Figure 2, acts as the cavity resonator sensor on the surface of which the biological antibody material is immobilized. The patch is placed alongside a small rectangular co-planar ground plane [23,24]. Figure 2 illustrates the antenna geometry on an alumina substrate with a dielectric constant of 9.6 and thickness *H_sub_*. The complete dimensions are shown in Table 2. The antenna surface is gold-plated with a thickness of 6 nm over nickel-chrome with a thickness of 6 μm by using the vacuum-coating machine Edwards E306 to prevent an interaction between the antenna (as shown in Figure 3a) and any chemical material used, while Figure 3b compares the measured and simulated reflection coefficient. The resonating antenna with a deposited layer of normal blood serum is considered the reference of the measurements from which the changes due to the viral layer’s deposition are measured. Preliminary measurements are done for the reflection coefficient and resonance frequency by using a portable VNA N9918A. Our reference is taken to be the antenna with a layer of normal blood serum deposited on it, from which the changes due to the viral layer’s deposition are measured. Figure 3b shows the reflection coefficient against frequency in the frequency range of 1.5 up to 3 GHz for both the simulation and measured results for comparison. It should be noted that there is a discrepancy between the numerical and experimental results, which is attributed to many factors such as soldering the SMA connectors, fabrication tolerance, etc. Fortunately, the virus detection process does not depend on simulation results. Simulation takes place to obtain the most optimum dimensions of the sensors.

## 3. Addressable Immobilization of Corona Virus

**Figure 4:**
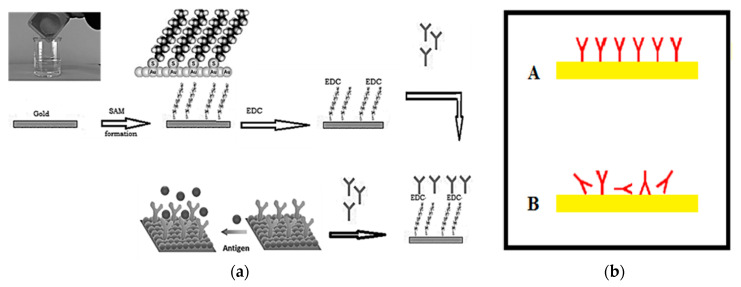
(**a**) Preparation and immobilization of antibodies on the gold surface of the sensor antenna, and (**b**) methods of immobilization of polyclonal antibodies on the microchip surface.

The SARS-CoV-2 strain (EPI_ISL_430820) and its specific antibodies were obtained from central laboratory of the Virus Research Center (the certified lab of coronavirus in Egypt) at the National Research Centre (NRC) [16]. The main objective of this task was to achieve the immobilization of SARS-CoV-2 virus antibodies in the right orientation (A) to yield stable antibodies mounted at the surface of the microchip, keeping the activity of specific Goat SARS-CoV-2 polyclonal antibodies at the highest and allowing layer growth when the infected sample was injected over the surface, as shown in Figure 4. This process can be summarized as follows: The gold slide was cleaned for 10 s in freshly prepared piranha solution: H_2_O_2_ (30%) 1 part and sulfuric acid (H_2_SO_4_, 96%) 3 parts. The gold slide was extensively washed with deionized water (Sigma Aldrich, St. Louis, MO, USA) then dried in a steam of nitrogen vapor.

Creating a SAM (self-assembled monolayer): a solution of 16-mercaptohexadecanoic acid (Sigma Aldrich, St. Louis, MO, USA) in ethanol (HPLC grade Merck, Kenilworth, NJ, USA, 200 micromolar solution) was prepared. The cleaned slides were completely immersed in the prepared solution and incubated for 8 h. Then, the slides were washed with ethanol and dried so that the surface was ready for antibody immobilization. Then, 30μg/mL polyclonal antibodies were circulated over the prepared surface for 60 min. Excess antibodies were then washed out by circulating HEPES solution, *N*-(2-Hydroxyethyl) piperazine-*N*′-(2-ethanesulfonic acid) (Merck-MFCD00006158, Sigma Aldrich, St. Louis, MO, USA).

**Figure 5:**
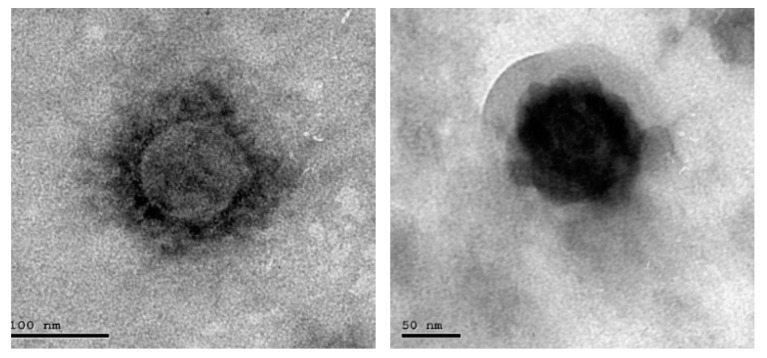
Photo of scanned COVID-19.

EDC Immobilization Method: 20 mg purified specific goat SARS-CoV-2 polyclonal antibody (Sigma Aldrich, St. Louis, MO, USA) was dissolved in 10 mL of 140 mM NaCl solution (Sigma Aldrich, St. Louis, MO, USA). Next, 500 mg of EDC (*N*-(3-Dimethylaminopropyl)-*N*-ethylcarbodiimide hydrochloride (Sigma Aldrich, St. Louis, MO, USA) was prepared in an Eppendorf tube. Then, we started pumping the solution of 140 mM NaCl through the cell, avoiding the formation of air bubbles at the surface of the chip. Then, we added the EDC to the polyclonal antibodies solution, stirred the solution vigorously active and pumped it immediately through the cell. We allowed the solution to circulate for 60 min, and then we washed the excess of unbound antibodies using NaCl solution for 10 min. The photo of the coronavirus used in our measurements was tested using a TEM (transmission electro-microscope) as shown in Figure 5.

## 4. System Integration and Assembly

**Figure 6:**
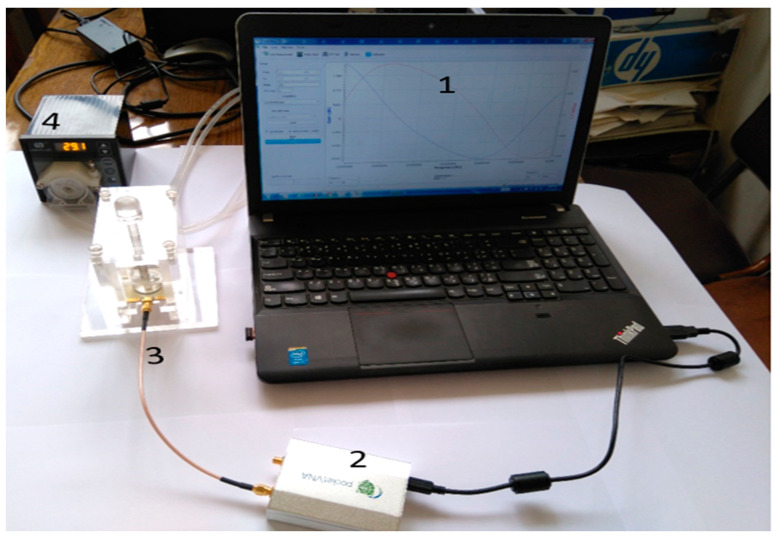
Components of the proposed microwave cavity resonator sensor system; No. 1 (laptop), No. 2 (VNA), No. 3 (3D housing of the sensor) and No. 4 (the micromechanical pump).

**Figure 7:**
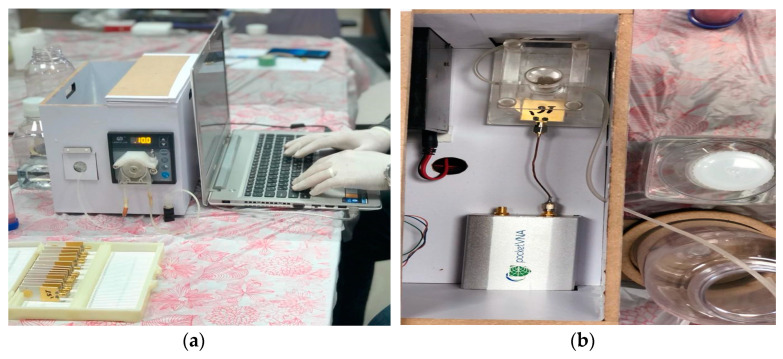
(**a**) Measurement setup for coronavirus samples and (**b**) proposed device inner view.

The proposed microwave cavity resonator prototype was used for the immobilized samples’ measurements. The measuring system prototype was as shown in Figure 6. Part No. 1 is the computer for automation software operation. Part No. 2 is the pocket vector network analyzer (VNA) for sample electrical characteristic measurements. Part No. 3 is manufactured 3D housing for the microwave sensor chip; the chip is inserted in its proper position in this 3D housing and a cavity circular room is adjusted over its surface. This cavity chamber is where the serum sample under testing passes. Part No. 4 is the micromechanical pump for the flow of liquids under test as well as the washing buffer. Micro-silicon tubes were used for inlets and outlets. The system was assembled in a laboratory prototype, as shown in Figure 6. Parts No. 2, 3 and 4 in Figure 6 were assembled as shown in Figure 7, while the laptop was used to run the control software and save the measuring results. The device has a weight of less than 1 kg and dimensions of 45 × 35 × 25 cm^3^. The laptop screen in Figure 6 illustrates the software home page. The device prototype is improved when shown in the experimental setup for the project of coronavirus sample measures shown in Figure 7a,b.

## 5. Coronavirus Samples Measured Results

**Figure 8:**
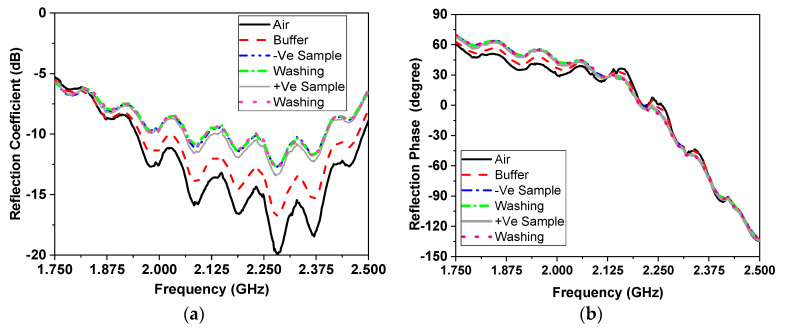
Reflection coefficient (**a**) phase for the samples and (**b**) magnitude when coated with COVID-19 at different concentrations.

The measurement results of the magnitude and phase of reflection coefficient of the tested samples are shown in Figure 8. Figure 8a shows the variation of S_11_ magnitude and shifting in resonant frequency by passing the serum over the sensor surface, while Figure 8b represents the change of phase of the sensor in degrees. In Figure 8, the black line is the sensor on board without any buffer, while the red dashed line is the sensor with a buffer to wash the surface. The −ve biological sample is the flow after that when the buffer is used for washing. The black thin line, meanwhile, is the +ve biological sample flow when the buffer is used for washing. This process was repeated 66 times for the 66 measured samples. Table 3 provides some sample of the measured results.

## 6. Statistical Analysis and Software Packages

Logistic regression analysis was used to predict or estimate the probability of infection using a patient’s sample. As multivariate regression analysis needs a relatively large sample size for either estimation or prediction; we used logistic regression analysis both for exploration and as a confirmatory method for coronavirus infection (in coronavirus, a total of 66 infected samples were measured). Logistic regression is a type of regression analysis where the outcome variable is binary (infected versus non-infected) and the predictor(s) or explanatory variables could be any type of variable [32,33]. Logistic regression, Equation (1), takes the following form:

(1) $$P(y|x)=\frac{e^{\alpha +\beta 1x1+\beta 2x2}}{1+e^{\alpha +\beta 1x1+\beta 2x2}}$$

The parameters in the equation include: α = constant derived from the analysis; β = slope for the independent variable(s) (*predictors* *Xi*); *P* (*y*|*x*) = probability of having the infection (range of 0 to 1) with a cutoff value of 0.5 or more indicating infection.

SPSS (Statistical Package for Social Sciences) version 23.0 (Chicago, IL, USA) was used for data analysis. Receiver operator characteristic curve (ROC) analysis was used to define a cut-off level for the three parameters that show changes in the electrical properties of organisms (before, after and as an absolute difference in values), namely:

1- Resonance frequency (GHz)
2- Magnitude of reflection coefficient S_11_ (dB)
3- Phase of reflection coefficient S_11_ (°)

Outcome values that define the discriminating ability of the parameter using ROC analysis include:

1- Sensitivity: the probability of the test producing +ve results if the patient is infected
2- Specificity: the probability of the test producing −ve results if the patient is not infected
3- Predictive value of positive test: the probability that the patient will have an infection if the test result is positive
4- Predictive value of negative test: the probability that the patient will not have an infection if the test result is negative
5- Total accuracy: the combined discriminatory results of the test for both true positive and true negative results

**Figure 9:**
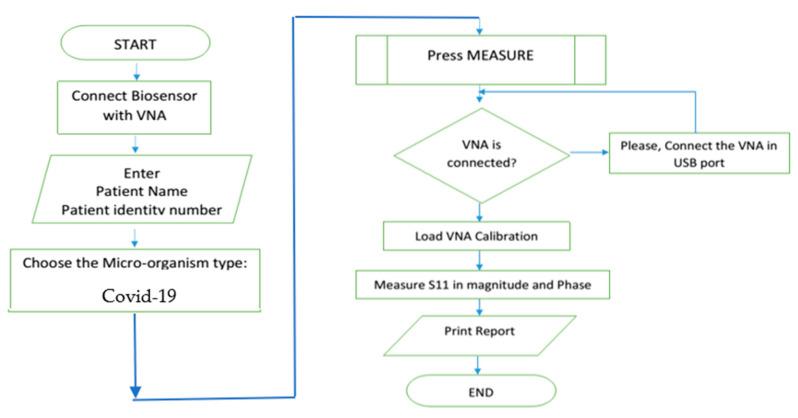
Flow chart of the proposed software.

According to different aims, the ROC analysis is useful to evaluate the discriminatory ability of a continuous marker (e.g., S_11_) to correctly assign a result into a two-group classification, find an optimal cut-off point to least misclassify the two-group subjects, compare the efficacy of two (or more) diagnostic tests or markers, and study the inter-observer variability when two or more observers measure the same continuous variable [33]. The accuracy depends on sensitivity, specificity, positive prediction values and negative prediction values. System control software is developed for automation with security over the manipulated data as shown in next flowchart, Figure 9.

## 7. Measured Samples and Measurement Methods

A total of 66 samples were measured using the proposed biosensor for validation and proof of concept as shown in Table 4. The measurement method is summarized as follows for both −ve and +ve tests done of the anti-COVID-19 polyclonal antibody.

### 7.1. Results of Measured Resonating Frequency

**Figure 10:**
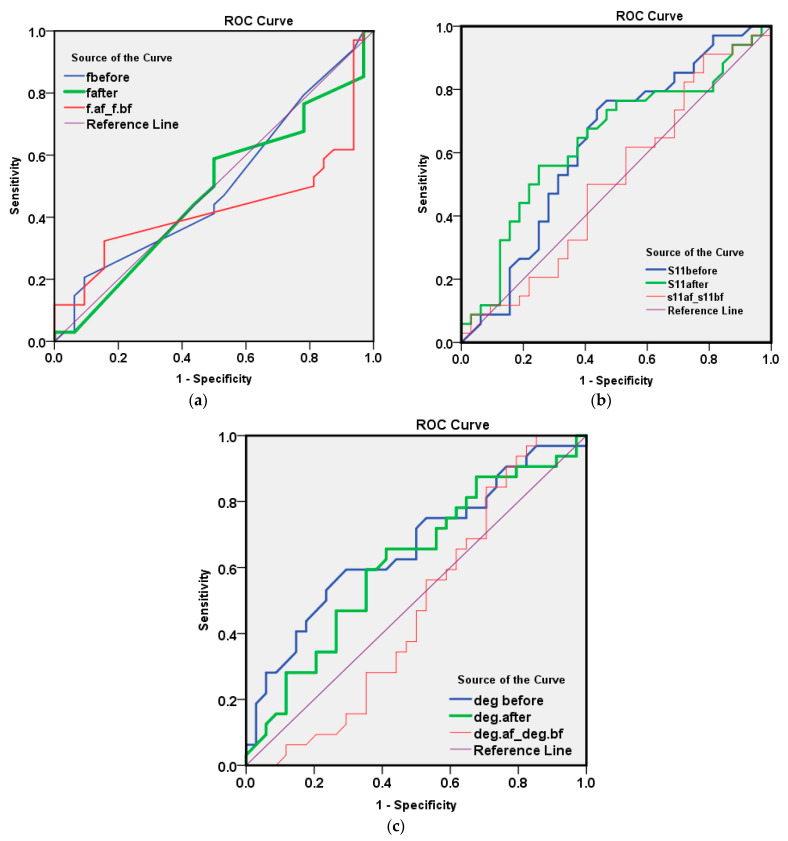
ROC method results for the 66 measured samples with respect to parameter one of the (**a**) resonating frequency, (**b**) reflection coefficient amplitude and (**c**) reflection coefficient phase in degrees.

An illustration of the detection results depending on the first measured parameter of resonating frequency using ROC analysis is shown in Figure 10 and Table 5 using f_before_, f_after_ and f_difference_. The area under the curve that discriminates infected from non-infected cases was close to null (area = 0.5) for the three f parameters, as shown in the above table, and the *p* values were all non-significant.

### 7.2. Results of Measured Reflection Coefficient Amplitude

The illustration of the detection results depending on the second measured parameter of reflection coefficient amplitude at a resonating frequency when using ROC analysis are shown in Figure 10b and Table 6.

The AUC for S_11_ after washing (=0.640, *p* value = 0.051) was of a moderate value for discriminating infected COVID-19 from non-infected cases as shown in Table 7. By examining the co-ordinate of the curve to select a cut-off value for the best overall accuracy, we found the following results:

It was found that a reflection coefficient amplitude S_11_ after a value <−12.5 (positive cases has more negative values) has both a sensitivity and predictive value that are positive for about 65% of a cases, and both a specificity and NPV of 62.5%, with a total accuracy of 63.6%. This result is satisfactory and in the range of the accuracy of the commercially available methods for COVID-19 detection, such as the PCR method, with merits of the newly proposed method and device as presented above.

### 7.3. Results of Measured of Reflection Coefficient Phase in Degrees

The illustration of the detection results depending on the third measured parameter of reflection coefficient phase using ROC analysis are as shown in Figure 10c, Table 8 and Table 9.

The AUC for the reflection coefficient phase in degrees before washing (=0.662, *p* value = 0.024) was of a moderate value for discriminating infected COVID-19 from non-infected cases. By examining the co-ordinate of the curve to select a cut-off value for the best overall accuracy, we found the following:

The value of the reflection coefficient phase in degrees before a value <−20.0 (+ve cases have more negative values) had a sensitivity of only 50%, though a better specificity of 71.9%. The predictive positive value of the test was 65.4%, with an NPV of 57.5% and a total accuracy of 60.6%. Again, these parameters’ results were satisfactory and in the range of the commercially available methods for COVID-19 detection, such as the PCR method. The illustrated measurement results presented a double-check for measured results for the microwave cavity resonator biosensor against the values of the reflection coefficient amplitude and reflection coefficient phase. Accordingly, these two parameters gave an overall detection accuracy of 63.6% and 60.6%., respectively. These values were extracted from a small number of measurement samples (66 in total). These results were acceptable and verified the proposed method and device for detection of coronavirus infection. When comparing it to the commercially available methods, our method does not use any amplification cycles as are necessary in RT-PCR.

## 8. Conclusions

This paper described rapid coronavirus detection, which is an effective method to distinguish between the blood serum with and without COVID-19 infection. The proposed sensor was simulated using a 3D full-wave electromagnetic simulator. The proposed device operates at an ISM 2.45 GHz frequency range. The device consists of a 3D housing, which contains the sensor and the microwave cavity, in addition to a fluidic micro-pump, pocket VNA, silicon tubes and software that depends on the ROC biostatical method. This setup gives the required results within a few minutes. It has the advantage of a low cost, small size and light weight. In addition, the sensor does not need trained technicians and may be used at ports. Beyond these advantages, the predictive accuracy of the device is 63.3% and 60.6% for reflection and phase, respectively, which are in the range of commercially available methods for COVID-19 detection, such as the RT-PCR method. So, we can consider the two methods to be commercially comparable. However, the proposed technique provides real-time results with merits of portability and easy use.

## Figures and Tables

**Table 1:** Different diagnostic techniques for detection of COVID-19 [14].

Diagnostic Tech.	Detection	Type of Sample	Laboratory/Point of Care Test	Advantage	Disadvantage	Size	Ref.
RT-PCR	Viral RNA	Nasopharyngeal swab, sputum, stool	Laboratory based	Specific detection and time saving	Low viral load gives false negative results.	large	[14]
Serology	Antibody/antigen	Blood	Laboratory based/point of care	Less complex than molecular tests	The antibodies may react with other pathogens in addition, giving false negative results for low viral loading.	large	[15]
ELISA	Antibody	Blood	Laboratory based	Simple and cheap laboratory technique, important for vaccine	ELISA tests have not been matured yet.	medium	[16]
MCR Proposed	Antibody/antigen	Blood	Laboratory based	Moderate price and fast results	May give a false negative when the virus concentration is low.	small	[18]

**Table 2:** Dimensions of the proposed sensor antenna in mm.

*L_g_*	*Lf*	*L_sub_*	*R*	*W_sub_*	*Tan δ*	*W_f_*	*L_s_*	*H_sub_*
17	42	50	6.5	25	0.0035	3	10	1.28

**Table 3:** Examples of the shift in resonating frequency and phase before and after washing of samples.

COVID-19	Reflection Coefficient				Phase	
Serial	f_before_ (GHz)	f_after_ (GHz)	S_11before_ (dB)	S_11after_ (dB)	deg _before_ (°)	deg _after_ (°)
1	2.285	2.285	−12.44985	−12.0007	−26.25539	−25.62203
2	2.285	2.2775	−12.0007	−12.12683	−25.62203	−20.1454
3	2.285	2.285	−10.85233	−10.84041	−16.72242	−17.47174
4	2.285	2.37	−10.85233	−10.67627	−16.72242	−67.83884
5	2.285	2.285	−12.14496	−11.82519	−23.64535	−25.88884
6	2.285	2.275	−11.82519	−11.7540	−25.88884	−17.6381
7	2.285	2.285	−16.03847	−15.6323	−19.85438	−21.78697
8	2.285	2.29	−15.63235	−15.3149	−21.78697	−26.52782
9	2.285	2.285	−11.39496	−11.2455	−15.00481	−16.92356
10	2.285	2.2825	−11.24558	−10.9067	−16.92356	−16.62737
11	2.285	2.285	−11.93321	−11.9472	−24.97691	−22.87379
12	2.285	2.29	−11.94724	−11.7377	−22.87379	−27.84187
13	2.285	2.2875	−12.61087	−11.9033	−24.24766	−28.77753
14	2.2875	2.2825	−11.90339	−11.8353	−28.77753	−11.92937

**Table 4:** Final number of COVID-19 samples.

	Frequency	Percent
−ve COVID-19	32	48.5
+ve COVID-19	34	51.5
Total	66	100.0

**Table 5:** Area under the curve for the analysis results of the first measurement parameter of the resonating frequency.

Test Result Variable(s)	Area	Std. Error	Asymptotic Sig.	Asymptotic 95% Confidence Interval	
				Lower Bound	Upper Bound
f_before_	0.501	0.072	0.985	0.360	0.642
f_after_	0.480	0.072	0.783	0.340	0.621
f_after_-f_before_	0.430	0.074	0.330	0.284	0.576

**Table 6:** Area under the curve for the analysis results of the second measurement parameter of the reflection coefficient amplitude.

Test Result Variable(s)	Area	Std. Error	Asymptotic Sig.	Asymptotic 95% Confidence Interval	
				Lower Bound	Upper Bound
S_11before_	0.626	0.070	0.079	0.488	0.764
S_11after_	0.640	0.069	0.051	0.504	0.776
S_11after_-S_11before_	0.511	0.072	0.878	0.369	0.653

**Table 7:** The analysis results of the measurement parameter.

	Sensitivity (<−12.5)	Specificity (>−12.5)	PPV	NPV	Total Accuracy
S_11_ after value cutoff = −12.5	22/34 (64.7%)	20/32 (62.5%)	22/34 (64.7%)	20/32 (62.5%)	44/66 (63.6%)

**Table 8:** Area under the curve for the analysis results of the third measurement parameter of the reflection coefficient phase.

Test Result Variable(s)	Area	Std. Error	Asymptotic Sig.	Asymptotic 95% Confidence Interval	
				Lower Bound	Upper Bound
deg _before_	0.662	0.068	0.024	0.529	0.794
deg _after_	0.615	0.070	0.109	0.478	0.752
deg _after_-deg _before_	0.474	0.073	0.719	0.332	0.617

**Table 9:** The analysis results of the measurement parameter.

	Sensitivity (<−20.0)	Specificity (>−20.0)	PPV	NPV	Total Accuracy
deg _before_ value cutoff = −20.0	17/34 (50%)	23/32 (71.9%)	17/26 (65.4%)	23/40 (57.5%)	40/66 (60.6%)

## Data Availability

The datasets generated during and analyses during the current study are available from all authors on reasonable request.
